# Gene Ontology annotations: what they mean and where they come from

**DOI:** 10.1186/1471-2105-9-S5-S2

**Published:** 2008-04-29

**Authors:** David P Hill, Barry Smith, Monica S McAndrews-Hill, Judith A Blake

**Affiliations:** 1 The Jackson Laboratory, Bar Harbor, ME, USA; 2 Department of Philosophy and Center of Excellence in Bioinformatics and Life Sciences, University at Buffalo, NY, USA

## Abstract

To address the challenges of information integration and retrieval, the computational genomics community increasingly has come to rely on the methodology of creating annotations of scientific literature using terms from controlled structured vocabularies such as the Gene Ontology (GO). Here we address the question of what such annotations signify and of how they are created by working biologists. Our goal is to promote a better understanding of how the results of experiments are captured in annotations, in the hope that this will lead both to better representations of biological reality through annotation and ontology development and to more informed use of GO resources by experimental scientists.

## Background

The PubMed literature database contains over 15 million citations and it is beyond the ability of anyone to comprehend information in such amounts without computational help. One avenue to which bioinformaticians have turned is the discipline of ontology that allows experimental data to be stored in such a way that it constitutes a formal, structured representation of the reality captured by the underlying biological science. An ontology of a given domain represents types and the relations between them, and is designed to support computational reasoning about the instances of these types. From the perspective of the biologist, the development of bio-ontologies has enabled and facilitated the analysis of very large datasets. This utility comes not from the ontologies per se, but from the use to which they are put during the curation process that results in ‘annotations’.

The principal use of an ontology such as the GO [1] is for the creation of annotations by the curators of model organism databases [e.g., [2-4]] and at genome annotation centers [5] who are striving to capture, in a form accessible by computational algorithms, information about the contributions of gene products to biological systems as reported in the scientific literature. Because such annotations are so integral to the use of bio-ontologies, it is important to understand how the curatorial process proceeds. We demonstrate here how the GO annotation paradigm illustrates important aspects of this process.

To help in understanding this work, we provide a glossary of the terms that are most important to our discussion:

An ***annotation*** is the statement of a connection between a type of gene product and the types designated by terms in an ontology such as the GO. This statement is created on the basis of observations of the instances of such types made in experiments and of the inferences drawn from such observations. For present purposes we are interested in the annotations prepared by model organism databases to a type called ‘gene’, a term which is seen as encompassing all gene-product types. For the purpose of this discussion, we do not need to address the distinction between gene and gene product.

An ***instance*** is a particular entity in spatio-temporal reality, which instantiates a type (for example, a type of gene product molecule, a type of cellular component). In the cases discussed here, the instances would be actual molecules or cellular components that can be physically identified or isolated or associated biological processes that can be physically observed.

A ***type*** (aka “universal”) is a general kind instantiated by an open-ended totality of instances that share certain qualities and propensities in common. For example, the type *nucleus*, whose instances are the membrane bound organelles containing the genetic material present in instances of the type *eukaryotic cell*.

A ***level of granularity*** is a collection of instances (and of corresponding types) characterized by the fact that they form units (‘grains’), such as molecules, cells, organisms in the organization of biological reality. Successive levels of granularity form a hierarchy by virtue of the fact that grains at smaller scales are parts of grains at successively larger scales.

A ***gene product instance*** is a molecule (usually an RNA or protein molecule) generated by the expression of a nucleic acid sequence that plays some role in the biology of an organism. For example, an instance of the *Shh* gene product would be a molecule of the protein produced by the *Shh* gene.

A ***molecular function instance*** is the enduring potential of a gene product instance to perform actions, such as catalysis or binding, on the molecular level of granularity. A molecule of the *Adh1* gene product sitting in a test tube has the potential to catalyze the reaction that converts an alcohol into an aldehyde or a ketone. It is assumed that in the correct context, this catalysis event would occur. The potential of this molecule describes its molecular function.

A ***biological process instance*** (aka “occurrence”) is a change or complex of changes on the level of granularity of the cell or organism that is mediated by one or more gene products. For example, the development of an arm in a given embryo would be an instance of the biological process *limb development*.

A ***cellular component instance*** is a part of a cell or its extracellular environment where a gene product may be located. For example, a cellular component instance *intrinsic to internal side of plasma membrane* is that part of a specific cell that comprises the lipid bilayer of the plasma membrane and the cytoplasmic area adjacent to the internal lipid layer where a gene product would project.

For each of the *instance* terms in the above, there is a corresponding *type* term defined in the obvious way; thus a *molecular function type* is a type of molecular function instance, and so on.

***Curation*** is the creation of annotations on the basis of the data (for example data about gene products) contained in experimental reports, primarily as contained in the scientific literature published on the basis of the observation of corresponding instances.

An ***evidence code*** is a three-letter designation used by curators during the annotation process that describes the type of experimental support linking gene product types with types from the GO Molecular Function, Cellular Component and Biological Process ontologies. For example, the evidence code ***IDA*** (Inferred from Direct Assay) is used when an experimenter has devised an assay that measures the execution of a given molecular function and the experimental results show that instances of the gene product serve as agents in such executions. An assay is designed to detect, either directly or indirectly, those occurrences that are the executions of a given molecular function type. Thereby the assay identifies instances of that function type. The code ***IGI*** (Inferred From Genetic Interaction) is used when an inference is drawn, from genetic experiments using instances of more than one gene product type, to the effect that molecules of one of these types are responsible for the execution of a specified molecular function.

The Gene Ontology Consortium (GOC) uses two further evidence codes to describe experimental support for an annotation: ***IMP*** (Inferred by mutant phenotype), and ***IPI*** (Inferred by physical interaction). The consortium uses other evidence codes to describe inferences used in annotations that are not supported by direct experimental evidence, but these will not be considered in this discussion (). Here we give examples of the process of annotation supported by experimental evidence using the IDA and IMP evidence codes. We use these examples to illustrate how using an annotation helps us understand the underlying biological methods that were used to support the inferences between the types that the annotation represents. With this knowledge in hand, we can then use this information to generate new inferences or to filter the information for specific needs.

## Results

### The curator perspective

**Figure 1:**
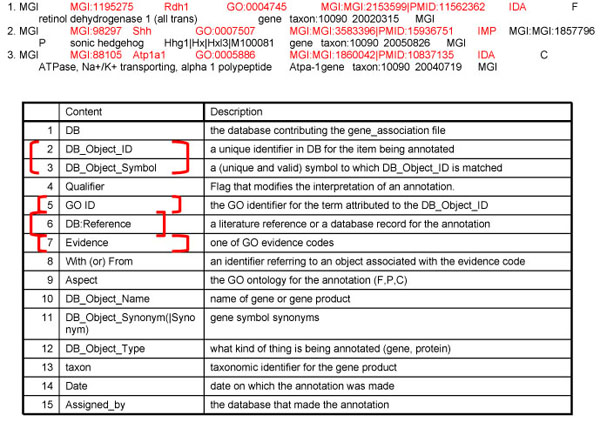
**Anatomy of an Annotation.** Annotations are provided to the Gene Ontology Consortium as tab-delimited files with 15 fields. Four fields indicate the gene product being annotated, the ontology terms used in the association, the type of evidence supporting the annotation and the reference where the original evidence was presented. The three annotations described in this manuscript are shown.

A GO annotation represents a link between a gene product type and a molecular function, biological process, or cellular component type (a link, in other words, between the gene product and what that product is capable of doing, what biological processes it contributes to, and where in the cell it is capable of functioning in the natural life of an organism). Formally, a GO annotation consists of a row of 15 columns. For the purpose of this discussion, there are 4 primary fields: i) the public database ID for the gene or gene product being annotated ; ii) the GO:ID for the ontology term being associated with the gene product; iii) an evidence code, and iv) the reference/citation for the source of the information that supports the particular annotation (Figure 1). Curators from the GOC have agreed to use standard practices when annotating gene products, practices are enforced by e-mail exchanges, quality control reports, face-to-face meetings and regular conference calls.

Additional details of these practices and of the annotation structure and GO-defined annotation processes are available at the GO website []. Briefly, the annotation process unfolds in a series of steps. First, specific experiments, documented in the biomedical literature, are identified as relevant to the curation-process responsibilities of a given curator. Second, the curator applies expert knowledge to the documentation of the results of each selected experiment. This process entails determining which gene products are being studied in the experiment, the nature of the experiment itself, and of the molecular functions, biological processes and cellular components that the experiment identifies as being correlated with the gene product. The curator then creates an annotation which captures the appropriate relationships between the corresponding ontology types.

Finally, annotation quality control processes are employed to ensure that the annotation has a correct formal structure, to evaluate annotation consistency among curators and curatorial groups, and to harvest the knowledge emerging from the activity of annotation for the contributions it might make to the refinement and extension of the GO itself, and increasingly also to other ontologies.

*Step 1: Identification of relevant experimental data*: The main goal of the GO annotation effort is to create genome-specific annotations supported by evidence obtained in experiments performed in the organism being annotated. However, many annotations are inferred from experiments performed in other organisms, or they are inferred not from experiments at all but rather from knowledge about sequence features for the gene in question. Such information, too, is captured in the GO annotations by means of corresponding evidence codes. It is thus important for the user of such annotations to understand what these codes reflect either that an annotation is based on experimental evidence supporting the assertion or that an annotation is a prediction based on structural similarity. The difference between experimentally verified and computationally derived GO annotations can be identified in the annotation file. This complexity, if not taken into account by the user, can confound data analyses and undermine the goal of hypothesis generation on the basis of GO annotation sets. With an understanding of the kinds of evidence that underlie a given GO annotation and of how that annotation is meant to represent the real world, the user can intelligently filter annotation files and retrieve those annotation sets that reflect the kinds of experiments and of predictions that are of maximal relevance.

*Step 2: Identification of the appropriate ontology annotation term:* The decision as to what GO term to use in an annotation depends on several factors. The experiment itself will bring some limit on the resolution of what can be understood from its results. For example, cell fractionation might localize molecules of a protein to the nucleus of a cell, but immunolocalization experiments might localize molecules of the same type of protein to the nucleolus of a cell. As a result, the same gene may have annotations to different terms in the same ontology because annotations are based on different experiments. Efforts are made to ensure annotation consistency through regular annotation consistency checks. Where inconsistencies are identified, the GOC takes steps to resolve them by working with the curators involved and where necessary with domain specialists. The limitations of experimental methods may lead curators to use their own scientific expertise and background knowledge when selecting a term. It is important to keep in mind that the choice of a GO term is sometimes made by inference made by the annotator on the basis of his or her previous knowledge. An example would be the case in which a mutation in a housekeeping gene causes a defect in a very broad process such as limb morphogenesis. A curator who has background knowledge about the function of this gene as being involved in basic cell physiology may be confident that the defect in morphogenesis is a by-product of unhealthy cells, and that the gene product is not involved in morphogenesis *per se*. The task of establishing which sub-processes are parts of and which lie outside a given process is challenging not only to ontology developers and curators but also to laboratory biologists. One method to address this issue is to define each process with a discreet beginning and end. GO ontology developers use this method whenever possible when defining process types. This allows annotators to best capture the knowledge based on the GO type defined. This GOC has now adopted a policy, already being realized by the MGI group, of creating annotations that are ‘contextual’. This means that terms from other ontologies such as the cell type (CL) (6) and other OBO Foundry ontologies (7), and from the mouse anatomical dictionary (8) are used in conjunction with GO terms in the annotations. As a result, the annotation can more accurately describe the biological reality that needs to be captured.

### Molecular function annotation

In the simplest biological situation, molecules of a given type are associated with a single molecular function type. A specific molecule *m* is an instance of a molecule type *M* (represented for example in the UniProt database), and its propensity to act in a certain way is an instance of the molecular function type *F* (represented by a corresponding GO term). So, a molecule of the gene product type *Adh1*, alcohol dehydrogenase 1 (class I), has as its function an instance of the molecular function type *alcohol dehydrogenase activity*. This means that such a molecule has the potential to execute this function in a given contexts. The term ‘activity’, in this sense, is meant as it is used in a biochemical context; and is more appropriately read as meaning: ‘potential activity’. Note that although the same string, “alcohol dehydrogenase”, is used both in the gene name and in the molecular function, the string itself refers to different entities: in the former to the molecule type; in the latter to the type of function that molecule has the propensity to execute. This ambiguity is rooted in the tendency to name molecules based on the functions they execute, and it is important to understand this distinction since the name of a molecule and the molecular function to which the molecule is attributed may not necessarily agree, for instance because the molecule may execute multiple functions.

If we say that instances of a given gene product type have a potential to execute a given function, this does not mean that every instance of this type will in fact execute this function. Thus molecules of the mouse gene product type *Zp2* are found in the oocyte and have the propensity to bind molecules of the gene product type *Acr* during fertilization [9]. If, however, an oocyte is never fertilized, the molecules still exist and they still have the propensity to execute the binding function, but the function is never executed.

**Figure 2:**
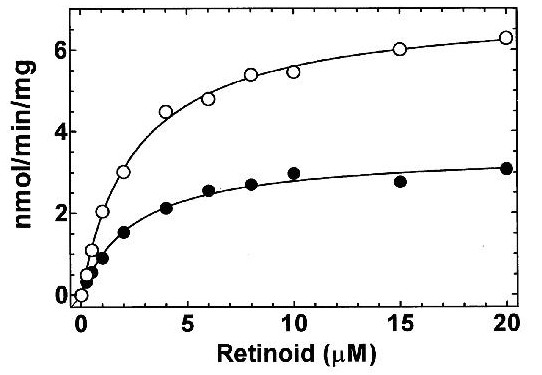
**Molecular Function Annotation Data**. This graph is reproduced from Zhang *et al* [10]. The graph shows the concentration of retinoid used as substrate along the X axis and the retinol dehydrogenase activity along the Y axis. Open circles refer to all-trans-retinol as a substrate and closed circles refer to 9-cis-retinol as a substrate. The enzyme samples were taken from a crude extract of cells transfected with a cDNA encoding the *Rdh1* gene. [Used by permission]

The experimental evidence used to test whether a given molecular function type *F* exists comes in the form of an ‘assay’ for the execution of that function type in molecules of some specific type *M*. If instances of *F* are identified in such an assay, this justifies a corresponding molecular function annotation asserting an association between *M* and *F*. As an example, Figure 2 shows results of an assay for the molecular function *retinol dehydrogenase activity* taken from a study by Zhang *et al*. [10] (Throughout this paper we will denote types using italics.) The molecular function type *retinol dehydrogenase activity* is defined in the molecular function ontology by the reaction: retinol + NAD^+^ → retinal + NADH + H^+^. Instances of gene product molecules annotated to this term have the potential to execute this catalytic activity. In this experiment, a cell protein extract was incubated with two substrates, all-trans-retinol (open circles) or 9-cis-retinol (filled circles), and the cofactor NAD^+^ for 10 minutes and the amount of retinal generated was measured. The graph shows the rate of accumulation of product (retinal) with respect to the concentration of substrate (retinoid) used. The results show that the reaction defined by the GO molecular function type *retinol dehydrogenase activity* has indeed been instantiated – the execution of this function has occurred. The observed occurrences of retinol being converted to retinal are evidence for the existence of instances of this molecular function type. In this experiment, the instances of the function type are identified through observation of actual executions. We assert that some molecules in this extract have molecular functions of type *retinol dehydrogenase activity* because occurrences of executions of instances of this type have been directly measured.

### Biological process annotation

A molecular function instance is the enduring potential of a gene product instance to act in a certain way. A biological process instance is the execution of one or more such molecular function instances working together to accomplish a certain biological objective. A biological process instance is at the cellular or organismal level of granularity what the execution of a function is at the level of the molecule. There is a relationship between molecular functions and biological processes. At this time this relationship is not represented explicitly in GO. From a gene annotation perspective, we are interested in going beyond the instance-instance relations at the cell- or organism-level, and in gaining the ability to infer type-type relations which link gene product types at the molecular level of granularity to process types at the level of the cell or organism. We are interested in the fact that molecules of a given gene product type can be associated with instances of a molecular function type (known or unknown) whose execution contributes to the occurrence of a biological process of a given type. Inferences about such type-type relations can be made because experiments are designed to test what transpires when specified biological conditions are satisfied in typical circumstances – circumstances in which, as a result of the efforts of the experimenter, disturbing events do not interfere. Experiments are designed to be reproducible and predictive, describing the instances that one would expect to find in biological systems meeting the defined conditions. If future experiments show that preceding experiments did not describe the intended typical situation, then the conclusions from the preceding experiments are questioned and may be reanalyzed and reinterpreted, or even rejected entirely, and the corresponding annotations then need to be amended accordingly.

Annotations in this way sometimes point to errors in the type-type relationships described in the ontology. An example is the recent removal of the type *seretonin secretion* as an is_a child of *neurotransmitter secretion* from the GO Biological Process ontology. This modification was made as a result of an annotation from a paper showing that serotonin can be secreted by cells of the immune system where it does *not* act as a neurotransmitter.

Associations between gene products and biological processes, too, can be detected experimentally. When instances of biological process type *P* are detected, either by direct observation or by experimental assay, as being associated with instances of a given gene product type *M*, then this justifies the assertion of that sort of association between *M* and *P* which is called a biological process annotation.

**Figure 3:**
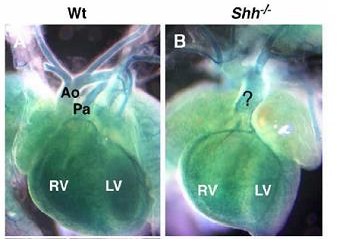
**Biological Process Annotation Data**. This figure is reproduced from Washington Smoak *et al* [11]. The figure shows micrographs of hearts in 16.5dpc mouse embryos. The figure on the left shows an animal with two functional copies of the *Shh* gene and the figure on the right shows an animal with no functional copies. Ao and Pa indicate the aorta and the pulmonary artery respectively. The ? indicates an aberrant outflow tract. Reprinted from Developmental Biology, 283, Washington Smoak *et al*, Sonic hedgehog is required for cardiac outflow tract and neural crest development, 357-72, Copyright 2005, with permission from Elsevier.

For those species of organisms where the tools of genetic study can be successfully applied, the association of gene product types with biological process types is usually achieved through the study of the perturbations of biological processes following genetic mutation. Curators use the IMP evidence code for these annotations. Figure 3 shows an example of a mutational analysis done by Washington-Smoak *et al* on the effects of a mutation of the *Shh* gene on mouse heart development [11]. The left panel shows an image of a heart with normal copies of the gene (WT) at 16.5 days of embryogenesis; the right panel shows a heart with defective copies of the gene at 16.5 days of embryogenesis. The figure clearly illustrates that the development of the outflow tracts of the heart is defective in the embryo with the defective gene. The GO Biological Process ontology defines the type *heart development* as: ‘the process whose specific outcome is the progression of the heart over time, from its formation to the mature structure. The heart is a hollow, muscular organ, which, by contracting rhythmically, keeps up the circulation of the blood.’

**Figure 4:**
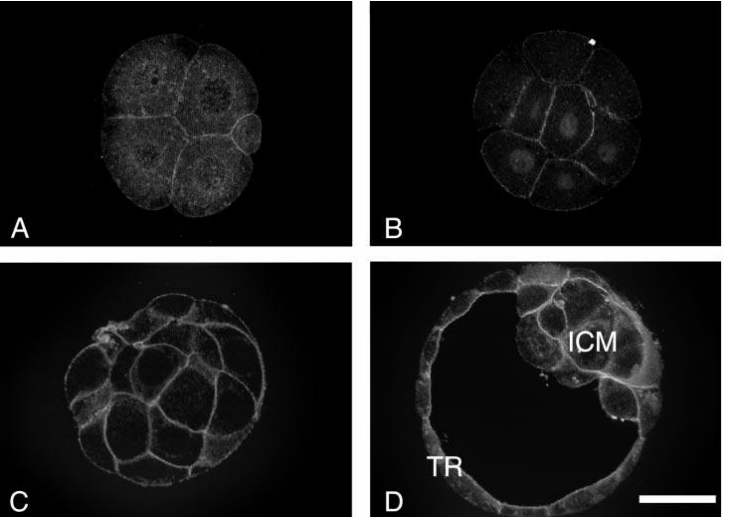
**Cellular Component Annotation**. This figure is reproduced from MacPhee *et al* [12]. The figure shows micrographs that are the results of an immunofluorescence localization of the ATP1A1 protein. The illuminated areas show the location of the protein along the plasma membrane. Reprinted from Developmental Biology, 222, MacPhee *et al*, Differential involvement of Na(+),K(+)-ATPase isozymes in preimplantation development of the mouse, 486-498, Copyright 2000, with permission from Elsevier.

Based on the mutational study reported in Washington-Smoak *et al*, an MGI curator has made an annotation linking *heart development* and the *Shh* gene using the IMP evidence code (Fig. 1). This annotation rests on the identification in the normal animal of a molecule of the product of the *Shh* gene with a molecular function whose execution contributes to an occurrence of the biological process *heart development*. We know that the biological process *heart development* exists because we observe it in the normal animal. We know that a molecule of SHH contributes to this process because when we take away all instances of the gene product of the *Shh* gene in an animal, the process of heart development is disturbed. The annotation thus affirms that a molecule of SHH protein has the potential to execute a molecular function that contributes to an instance of the type *heart development* in the Biological Process ontology. We also generalize that the execution of the molecular function of a molecule of SHH in a given mouse will in some way contribute to the development of that mouse's heart. However, the results of any phenotypic assay are limited to the resolution of the phenotype itself. In the experiment described above, we have validated the biological process, but cannot make any direct inferences about the nature of the function executed. It is for this and other practical reasons that the molecular function and biological process ontologies were developed independently.

### Cellular component annotation

In a large majority of cases, annotations linking gene product with cellular component types are made on the basis of a direct observation of an instance of the cellular component in a microscope, as for example in [12], which reports an experiment in which an antibody that recognizes gene products of the *Atp1a1* gene is used to label the location of instances of such products in preimplantation mouse embryos (Figure 4). The fluorescent staining shows that the gene products are located at the plasma membrane of the cells of these embryos. In this case, the instances of the gene products are the molecules bound by the fluorescent antibodies, and the instance of the cellular component is the plasma membrane that is observed under the microscope. A curator has accordingly used the results of this experiment to make an annotation of the *ATP1A1* gene product to the GO cellular component *plasma membrane* (Fig. 1). As with molecular functions and biological processes, there is also a relationship between molecular function and cellular component. It is straightforward to hypothesize that, if a molecule of a gene product is found in an instance of a given cellular component, then that gene product has the potential to execute its function in that cellular component as well. If the execution of the function is detected in the component, then we can make a generalization concerning the molecular function type and the cellular component type. We assume, based on the accumulated experimental data, that sufficient instances of the gene product will execute their functions in some instance of the cellular component type and that enough molecules will execute their function in such a way that these executions become biologically relevant. As with molecular function and biological process, experimental evidence for molecular function and cellular component annotations is often separable. Therefore, from a practical standpoint, these ontologies are also developed separately.

## Discussion

The development of an ontology for a given domain reflects a shared understanding of this domain on the part of domain scientists. This understanding, for biological systems, is the result of the accumulation of experimental results reflecting that iterative process of hypothesis generation and experimental testing for falsification which is the scientific method. The process of annotation brings new experimental results into relationship with the existing scientific knowledge that is captured in the ontology. There will necessarily be times when new results yield conflicts with the current version of the ontology. One of the strengths of the GO development paradigm is that development of the GO has been a task performed by biologist-curators who are experts in understanding specific experimental systems: as a result, the GO is continually being updated in response to new information. GO curators regularly request that new terms be added to the GO or suggest rearrangements to the GO structure, and the GO has an ontology development pipeline that addresses not only these requests but also submissions coming in from external users. By coordinating the development of the ontology with the creation of annotations rooted in the experimental literature, the validity of the types and relationships in the ontology is continually checked against the real-world instances observed in experiments. GO curators refer to this as annotation-driven ontology development. In addition, the GO community works with scientific experts for specific biological systems to evaluate and update GO representations for the corresponding parts of the ontology [13].

## Conclusions

Gene Ontology annotations report connections between gene products and the biological types that are represented in the GO using GO evidence codes. The evidence codes record the process by which these connections are established and reflect either the experimental analysis of actual instances of gene products or inferential reasoning from such analysis. We believe that an understanding of the role of instances in the spatiotemporal reality upon which experiments are performed can provide for a more rigorous analysis of the knowledge that is conveyed by annotations to ontology terms. While each annotation rests ultimately on the observation of instances in the context of a scientific experiment, the annotation itself is not *about* such instances. Rather it is about the corresponding types. This is possible because annotations are derived by scientific curators from the published reports of scientific experiments that describe *general cases*, cases for which we have scientific evidence supporting the conclusion that the instances upon which the experiments are performed are typical instances of the corresponding types. If such evidence is called into question through further experimentation, then as we saw, the corresponding annotations may need to be revised. The resultant tight coupling between ontology development and curation of experimental literature goes far towards ensuring that ontologies such as GO reflect the most sophisticated understanding of the relevant biology that is available to scientists. One area of future work would be to find ways to computationally identify inconsistencies in the type-type relations in the ontology based on inconsistencies of annotations to the types.

It is to us obvious that our cumulative biological knowledge should represent how instances relate to one another in reality, and that any development of bio-ontologies and of relationships between such ontologies should take into account information of the sort that is captured in annotations. While we are still at an early stage in the process of creating truly adequate and algorithmically processable representations of biological reality, we believe that the GO methodology of allowing ontology development and creation of annotations to influence each other mutually represents an evolutionary path forward, in which both annotations and ontology are being enhanced in both quality and reach.

## Competing interests

The authors declare that they have no competing interests.

## Authors' contributions

All authors contributed equally to this effort through discussion, writing, and revision of the manuscript.
